# Pelvic and central nervous system tuberculosis complicated by a paradoxical response manifesting as a spinal tuberculoma: a case report

**DOI:** 10.1186/s12879-022-07731-6

**Published:** 2022-09-24

**Authors:** Arya Zandvakili, Takaaki Kobayashi, Quanhathai Kaewpoowat, Meredith G. Parsons, Bradley Ford, Jason H. Barker, Melinda Johnson

**Affiliations:** 1 Division of General Internal Medicine, Department of Internal Medicine, University of Iowa Hospitals and Clinics, Iowa City, IA USA; 2 Division of Infectious Diseases, Department of Internal Medicine, University of Iowa Hospitals and Clinics, Iowa City, IA USA; 3 Department of Pathology, University of Iowa Hospitals and Clinics, Iowa City, IA USA

**Keywords:** Disseminated tuberculosis, Tuberculosis meningitis, Post-partum tuberculosis, Paradoxical reaction, Spinal tuberculoma

## Abstract

**Background:**

The post-partum period is a risk factor for tuberculosis (TB), possibly including the period after miscarriage as illustrated here. This case demonstrates how non-specific symptoms can hide widely disseminated TB.

**Case presentation:**

A healthy 26-year-old female with a history of recent miscarriage presented to the emergency department with non-specific symptoms of headache, abdominal pain, and sub-acute fevers. She had immigrated to the United States from the Marshall Islands 9 years prior. Two months prior to presentation she had a miscarriage at 18 weeks of pregnancy. On admission, transvaginal ultrasound revealed retained products of conception and abdominal computed tomography revealed findings consistent with tubo-ovarian abscesses and peritonitis. The obstetrics and gynecology service performed dilation and curettage (D&C) to remove retained products of conception. Acid-fast bacilli cultures from cerebrospinal fluid as well as specimens from D&C and intra-abdominal abscesses subsequently all grew TB. She was diagnosed with TB meningitis, peritonitis, endometritis, and tubo-ovarian abscesses. Her treatment course was complicated by a paradoxical response resulting in a spinal tuberculoma causing lower extremity weakness. The tuberculoma was treated with surgical decompression as well as continuation of treatment with anti-tubercular chemotherapy and steroids.

**Conclusion:**

Disseminated and extrapulmonary TB can present with non-specific symptoms. Recognition of risk factors for TB is critical for prompt diagnostic evaluation and treatment of this deadly disease. A paradoxical reaction needs to be taken into consideration when any new neurological symptoms occur during TB treatment.

## Background

Tuberculosis (TB) is a leading cause of morbidity and mortality with approximately 1.4 million deaths caused by TB in 2019 [1]. While TB is classically considered a respiratory illness, extrapulmonary TB (ETB) represents 15–20% of cases and often does not present with respiratory symptoms [1–4]. Therefore, ETB may be challenging to diagnose. Understanding the biological and social risk factors for ETB and its variations of presentation are critical for early diagnosis and prevention of complications.

Pregnancy is a relatively immunocompromised state, an adaptation that prevents maternal rejection of the fetus. Recent cohort studies have demonstrated an increased risk of TB during pregnancy and in the post-partum period [5, 6]. Though the exact mechanism of increased TB risk during pregnancy is unclear, it is thought that hormonal changes during pregnancy may alter activities of NK cells and T-cells [7, 8]. In addition, immune reconstitution in the post-partum period has been associated with unmasking or worsening symptoms of TB [9, 10]. While reports of TB presenting in the post-partum period are well documented [9–15], few have described TB presenting after abortion [16–19].

Here, we describe a case of TB meningitis, peritonitis, and endometritis in a healthy Marshallese woman presenting with non-specific symptoms of headache, abdominal pain, and sub-acute fevers after a second-trimester miscarriage. Her treatment course was complicated by a paradoxical response resulting in a spinal tuberculoma and lower extremity weakness.

## Case presentation

**Fig. 1:**
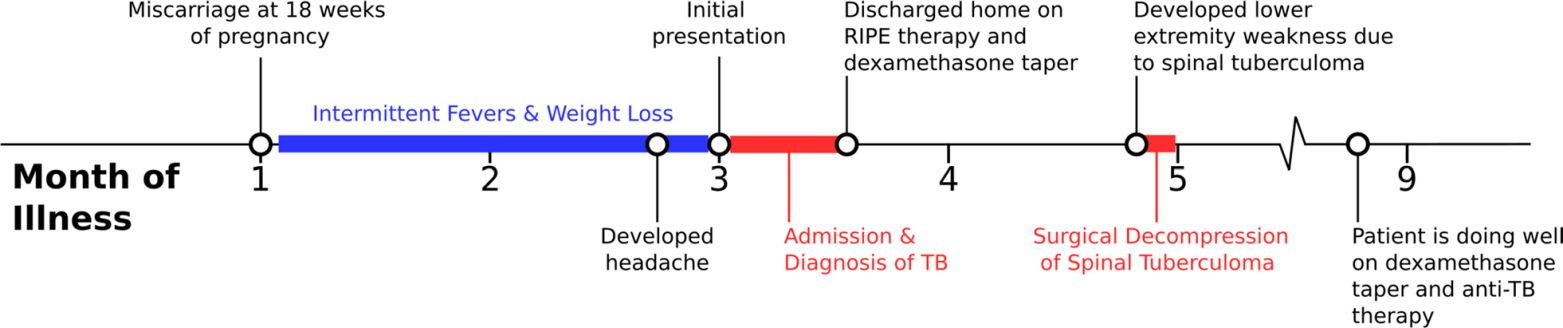
Timeline of the patient’s illness. *RIPE therapy* Rifampin, isoniazid, pyrazinamide, ethambutol

A healthy 26-year-old female with a history of recent miscarriage presented to the emergency department with 5 days of progressively worsening headache, neck pain, photophobia, and fatigue (Fig. 1). Four months prior to presentation she was found to be pregnant. Two months later (at 18 weeks of pregnancy) she experienced spontaneous vaginal bleeding and passing of the conceptus, followed by daily spotty vaginal bleeding and intermittent fevers. She did not consult her obstetrician. She experienced approximately 7 kg of weight loss during the month prior to presentation. Five days prior to presentation, she developed headache, neck pain, and fatigue. On the day of admission, she experienced what she described as “the worst headache of her life” and presented to the emergency department, where she denied cough, nausea, vomiting, diarrhea, rash, trauma, sick contacts, recent travel, and chronic illness. She was taking no medications. She worked as a housekeeper and had immigrated to the United States (US) from the Marshall Islands 9 years prior.

Vitals signs demonstrated temperature 38 °C, pulse 101 per minute, blood pressure 123/80 mmHg, and SpO2 96% on room air. Physical exam revealed nuchal rigidity and a diffusely tender abdomen. Laboratory workup revealed white blood cell count 6.7 × 10^3^/µl, hemoglobin 10.1 g/dL, and platelets 431 × 10^3^/µl. Computed tomography (CT) of the brain showed no intracranial hemorrhage, edema, midline shift, or mass effect. A lumbar puncture (LP) revealed clear cerebrospinal fluid (CSF) with glucose 19 mg/dL (reference range 40–75 mg/dL), protein 486 mg/dL (reference range 15–45 mg/dL), and white blood cell count 391 per mm^3^ (reference range 0–5 per mm^3^) with neutrophil predominance (60%). A BioFire FilmArray meningitis/encephalitis PCR panel (bioMerieux, Inc.) was negative. This panel tested for *Escherichia coli*, *Haemophilus*, *Listeria*, *Neisseria*, *Streptococcus agalactiae* and *pneumoniae*, *Cytomegalovirus*, *Enterovirus*, herpes simplex virus, human herpesvirus-6, human parechovirus, *Varicella*, and *Cryptococcus*. CSF was sent for aerobic and anaerobic culture. Bacterial meningitis was suspected; vancomycin and ceftriaxone were started empirically.

**Fig. 2:**
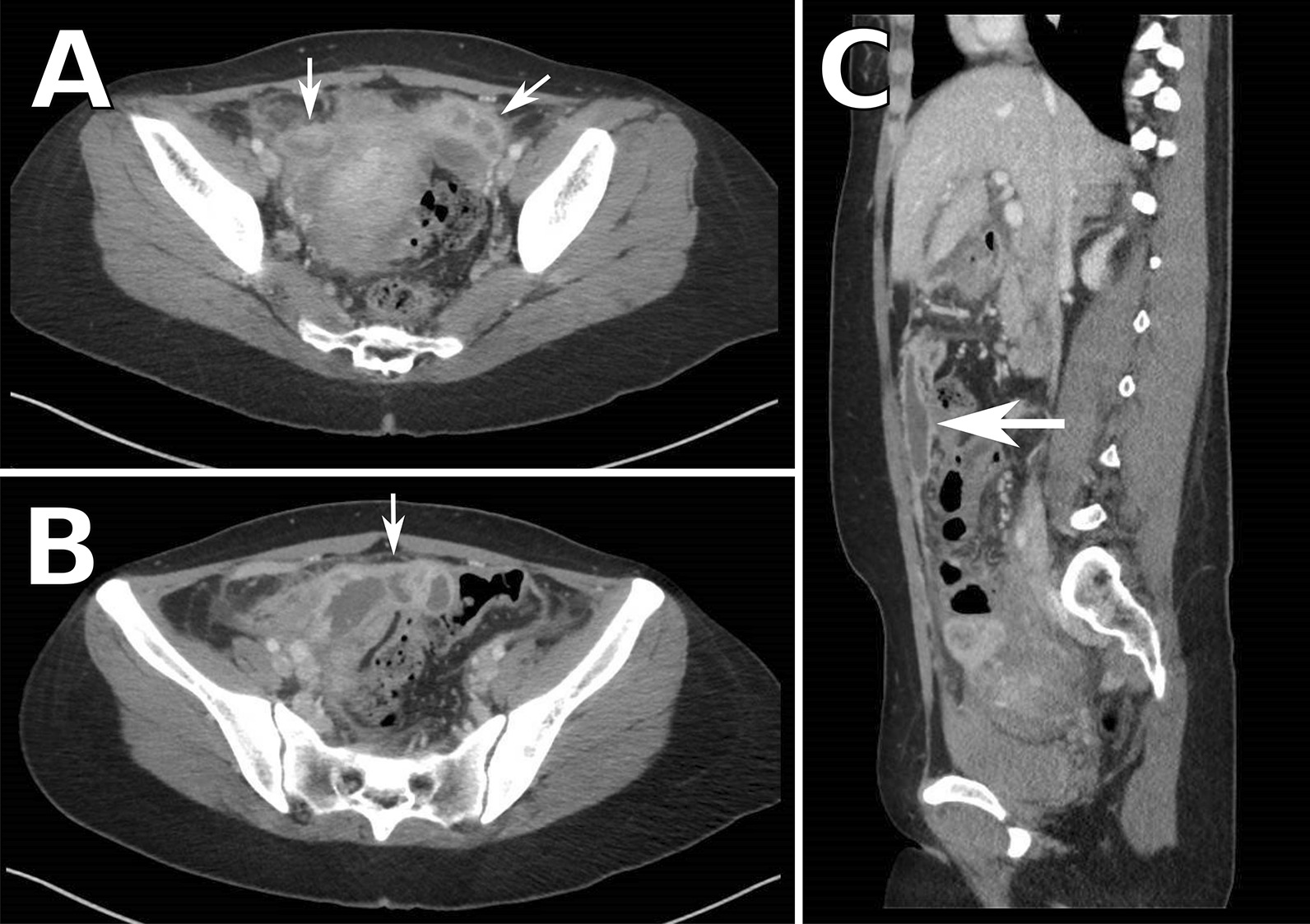
Computed tomography (CT) of abdomen/pelvis demonstrating tubo-ovarian and peritoneal abscesses.** A** Dilated, fluid-filled fallopian tubes with rim enhancement and thickening with **B** an associated tubo-ovarian abscess. **C** Peritoneal abscesses in the anterior mid and left mid-abdomen and pelvis

Given the patient’s recent miscarriage, abdominal pain, and continued vaginal bleeding, a transvaginal ultrasound was ordered which revealed retained products of conception and a tubal mass that wrapped around both ovaries. Abdominal and pelvic CT demonstrated dilated, fluid-filled fallopian tubes with wall-enhancement consistent with tubo-ovarian abscess and a large abscess (9.8 × 4.3 cm) adjacent to the fallopian tubes (Fig. 2). Additionally, CT demonstrated abscesses in the anterior mid-abdomen (6.7 × 1.3 cm) and left mid-abdomen (3.7 cm) as well as diffuse peritoneal thickening consistent with peritonitis. Doxycycline and metronidazole were added to cover anaerobes and common sexually transmitted bacteria associated with endometritis.

**Fig. 3:**
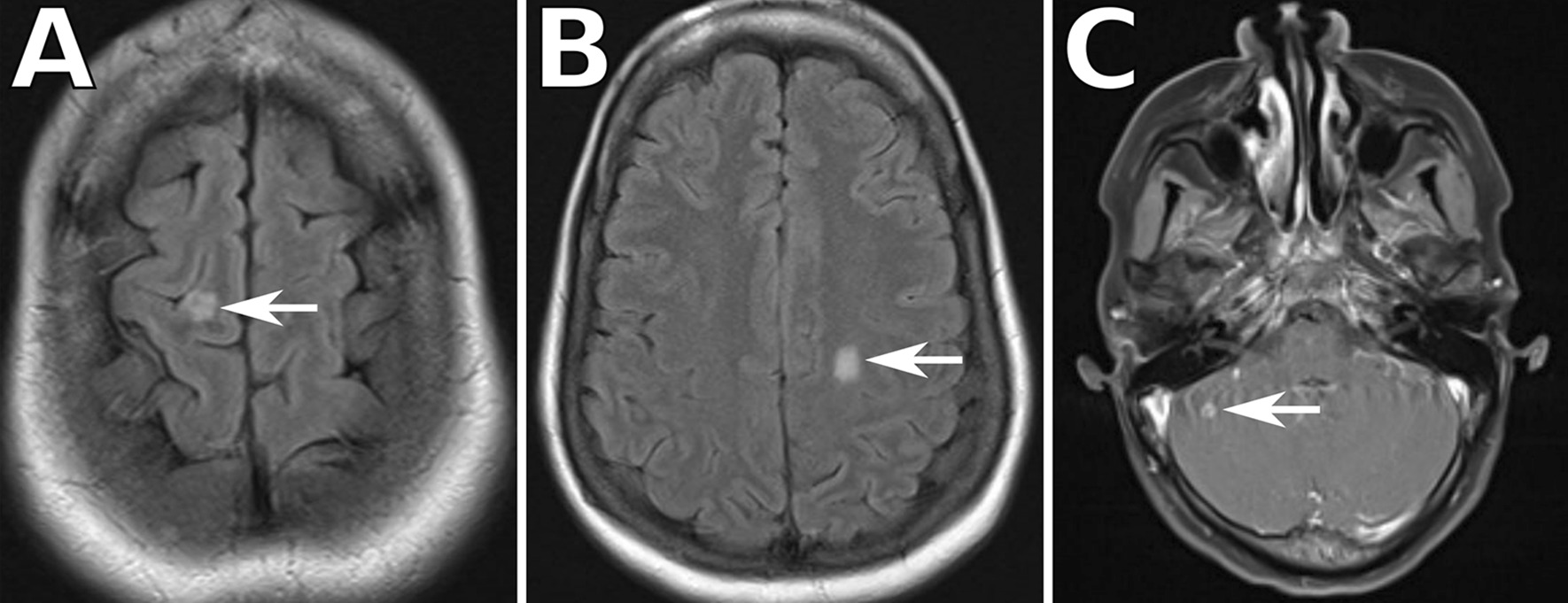
Magnetic resonance imaging of the brain showed **A**, **B** multiple supratentorial and **C** infratentorial enhancing lesions consistent with tuberculomas

On day 2 of admission the primary team consulted infectious disease specialists, who suspected tuberculous (TB) meningitis given the sub-acute course of illness and the patient’s origin from the Marshall Islands (a TB endemic region). Therefore, empiric treatment with rifampin, isoniazid, pyrazinamide, ethambutol (RIPE therapy), vitamin B6 (pyridoxine), and intravenous dexamethasone were started. She was placed in airborne isolation. Magnetic resonance imaging (MRI) of the brain revealed multiple supratentorial and infratentorial enhancing lesions, some showing rim enhancement, consistent with tuberculomas, with associated minimal leptomeningeal enhancement (Fig. 3). Her headache significantly improved with initiation of RIPE and dexamethasone, but did not resolve completely.

Also on day 2 of admission, the obstetrics and gynecology service performed dilation and curettage (D&C) to remove retained products of conception. Specimens were sent for histopathology as well as aerobic and anaerobic culture.

Chest X-ray appeared normal, but a chest CT demonstrated a consolidation in the left upper lung lobe suspicious for early TB. Three early sputum samples were negative for TB by PCR, AFB smear, and culture. A second LP was performed, and CSF was positive for TB by PCR on the Xpert MTB/RIF platform (Cepheid, Inc.).

**Fig. 4:**
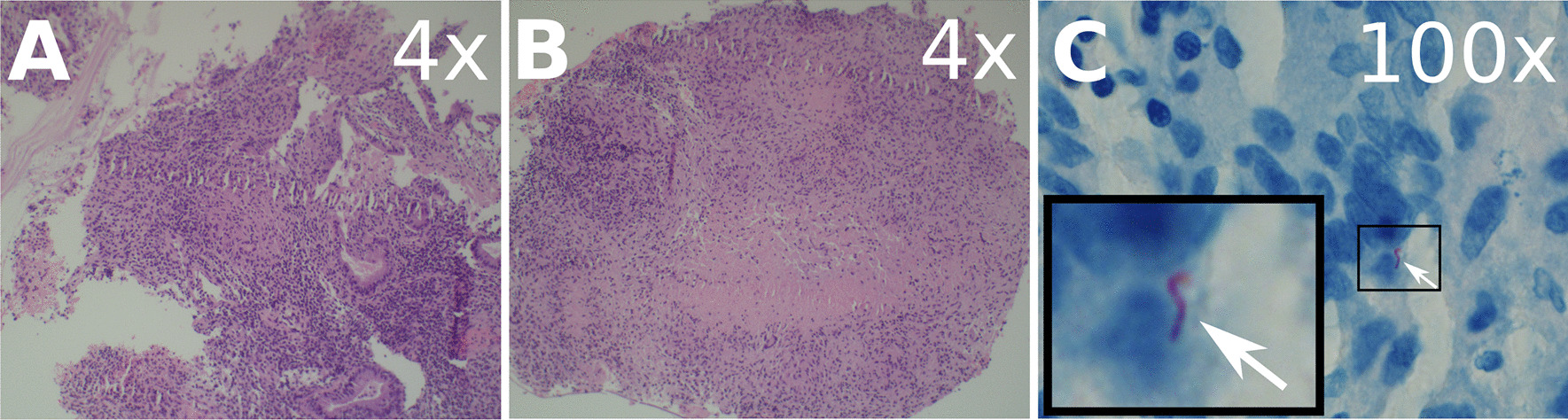
Stained endometrial biopsy sections. **A**, **B** Endometrial tissue with increased inflammation and necrotizing granulomas (H&E stain, 4× magnification). **C** Single acid-fast bacillus identified on histologic examination (AFB stain, 100× magnification). Inset image shows 467× magnified view of the acid-fast bacillus

On day 7, the interventional radiology service placed a drain in the tubo-ovarian abscess. Fluid from this drain was purulent and Gram-stain demonstrated many polymorphonuclear cells, but no organisms. At this point, it was not clear if the TB meningitis and abdominal/pelvic infections represented separate coincidental processes or a single disseminated process. She was maintained on RIPE and other antibiotics were narrowed to moxifloxacin since this agent has activity against both common abdominal/pelvic pathogens as well as TB. Histopathology of the endometrial specimen obtained during D&C eventually demonstrated necrotizing granulomatous inflammation with positive AFB stain (Fig. 4), prompting discontinuation of moxifloxacin.

As all her clinical symptoms including headache gradually improved, dexamethasone was replaced with oral prednisone daily. Unfortunately, this replacement resulted in a recurrence of headache. Therefore, therapy was switched back to dexamethasone with improvement of headache. Four days after drain placement in the tubo-ovarian abscess, a repeat CT demonstrated a significant decrease in abscess size, prompting drain removal.

On day 14 of hospitalization, the patient was discharged home with RIPE therapy, vitamin B6, and a 2-month taper of dexamethasone. After discharge, TB culture demonstrated pan-susceptibility, therefore ethambutol was discontinued. Eventually, all AFB cultures from the CSF, endometrial samples, and tubo-ovarian abscess grew *Mycobacterium tuberculosis*, indicating her entire disease could be attributed to disseminated TB.

**Fig. 5:**
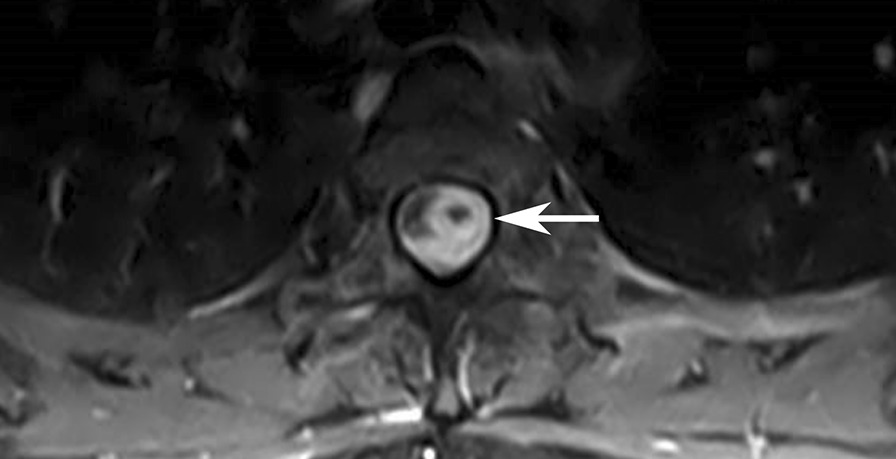
Magnetic resonance imaging showing a tuberculoma of the thoracic spine. Thoracic spine MRI showed an intradural extramedullary lesion at the level of T3/T4 invading the lateral aspect of the cord with severe mass effect and diffuse surrounding leptomeningeal enhancement throughout the thoracic spine. There was also high T2/STIR signal in the thoracic cord extending from T1-T7 levels concerning for cord edema

Unfortunately, five weeks after discharge she presented again to the emergency department with two days of progressively worsening distal paresthesia and left lower extremity weakness. She denied bowel or bladder incontinence. MRI of the spine demonstrated an intradural mass at the level of T3/T4, compressing the spine (Fig. 5). There was high T2-weighted/STIR signal extending from T1 to T7 concerning for cord edema. She was taken emergently to the operating room for decompression, requiring T3-T4 laminectomy and T5 partial laminectomy. In surgery, an intradural extramedullary tuberculoma was observed and washed out. AFB and fungal cultures of the mass remained negative. The cause of the spinal mass was suspected to be paradoxical TB reaction. Repeat spinal MRI showed increased cord T2-weighted/STIR signal at the surgical site. High dose dexamethasone was continued out of concern for persistent inflammatory changes in the spine as well as back pain. Pyrazinamide was discontinued after three months of treatment. Given the anticipated long duration of dexamethasone therapy, she was also prescribed trimethoprim-sulfamethoxazole for *Pneumocystis jiroveci* pneumonia (PJP) prophylaxis. It should be noted that current guidelines only recommend PJP prophylaxis for individuals receiving glucocorticoid doses equivalent to ≥ 20 mg of prednisone daily for one month or longer and have another immune-compromising condition [20]. At a 36-week follow-up appointment, she was taking isoniazid, rifampin, moxifloxacin, and dexamethasone and reported only mild back pain. Ultimately, her treatment duration will depend on her repeat spine MRI findings and her clinical course.

## Discussion and conclusions

This is a striking case of ETB with involvement of the CNS, abdominal cavity, and uterus presenting after miscarriage. Like similarly reported cases [9–19], it is likely that pregnancy increased the patient’s risk of reactivation of latent TB. Given the late presentation in this case, it is unclear whether TB caused the miscarriage or how changes in physiology (e.g., immune reconstitution) after the miscarriage contributed to the presentation.

Although meningitis represents only ~ 1% of TB cases in low prevalence areas [4, 21], its devastating consequences demand that it be promptly recognized and treated. Among HIV-negative patients, approximately 40% die or have severe disability (Rankin Scale 3 or greater) 9 months after presentation for TB meningitis [22, 23]. Early treatment is associated with reduction of poor outcomes [24, 25]. However, TB meningitis (TBM) is notoriously difficult to distinguish from typical bacterial meningitis, and in areas of low TB burden there is often a delay in diagnosis [25]. The Lancet scoring system was originally developed as criteria for diagnosing TBM for research purposes [26]. This scoring system is quite specific, but not sensitive, for distinguishing TBM from other causes of sub-acute meningitis [27]. On presentation, our patient would have scored 6 on the Lancet scoring system (classifying her as Possible TBM) due to weight loss (2 points) and CSF criteria (4 points). Once TBM is suspected, a nucleic-acid amplification test (NAAT) for TB on a CSF sample should be performed to aid in rapid diagnosis. Common NAAT methods, Xpert and Xpert Ultra (Cepheid Inc.), have near 100% specificity for TBM, but have only modest sensitivity (50–60%) for definitive TBM and are not FDA-approved for testing CSF so are not widely available for this purpose [28]. If TBM is suspected, empiric treatment should be initiated without waiting for TBM testing to result. Empiric treatment includes anti-tuberculous agents and adjunctive corticosteroids. Guidelines recommend a 2-month intensive phase with RIPE therapy followed by a continuation phase with only isoniazid and rifampin for 7–10 months [29–31].

Despite having disseminated TB with a pulmonary lesion, our patient’s sputum microscopy, culture, and PCR (Xpert MTB/RIF by Cepheid Inc) were negative for TB. While all three methods have greater than 90% specificity, they are not impervious to false negative results. Sputum microscopy is a rapid, cheap test that is widely available, but it is known to have low sensitivity. Conventional light microscopy has a sensitivity ranging typically from 50 to 75% and fluorescence microscopy has a sensitivity ranging typically from 50 to 95%. [32] Culture and PCR of sputum are significantly more sensitive for detecting pulmonary TB. A 2019 study in New York City found sputum culture to be 85% sensitive for pulmonary TB [33] and a meta-analysis of Xpert MTB/RIF performance found a sensitivity of 87–94% [34]. Hypothesized causes for false negative results include low mycobacterial burden in the sputum (especially in early pulmonary disease), recent use of antibiotics with anti-mycobacterial activity, or errors in sputum collection/testing. Given the non-negligible possibility for false-negative results, treatment for TB should be initiated when there is a strong suspicion for TB even if sputum is negative [29].

After initial improvement with anti-TB treatment, our patient had a paradoxical reaction (PR) with the development of an intradural extramedullary tuberculoma of the spinal cord. Rather than treatment failure, PR is thought to be a disproportionate inflammatory response after initiation of anti-TB therapy. While the exact mechanism is unclear, high antigenic load and immune-reconstitution after mycobacterial death have been proposed to drive the inflammatory response in PR [35]. PR is more common among TBM patients. A prospective cohort study of ~ 140 patients with TBM found PR occurred in about one-third of patients [36]. By comparison, another study found that only 4.5% of all TB patients develop a PR [37]. PR can be distinguished from treatment failure by demonstrating initial improvement of TB illness after initiation of anti-TB therapy for a significant period (e.g., 2 weeks) and absence of factors that reduce the efficacy of anti-TB therapy (e.g., resistance, poor adherence, malabsorption). PR typically occurs within 3 months of initiating anti-TB treatment, but it can present > 12 months after initiation of treatment [36, 38]. When PR is identified, anti-TB therapy should be continued and corticosteroid therapy should be either increased or re-initiated. TNF-α plays an important role in promoting the inflammatory response to TB and TNF-α antagonists have been proposed as a treatment for refractory PR [39].

TBM can be complicated by inflammation and infection of the spinal cord, resulting in radiculomyelitis, spinal tuberculoma, myelitis, syringomyelia, vertebral tuberculosis, or spinal tuberculous abscess [40]. Spinal involvement has been reported in 4–46% of patients with TBM with the median time to development of 3 months [41–43]. Focal neurological manifestations (e.g., weakness, paresthesias, radiculopathy, and bowel/bladder incontinence) are indicative of spinal cord compromise.

It is also important to recognize that our patient emigrated from the Republic of Marshall Islands (RMI), a Pacific Island nation where TB is endemic. In 2020, the incidence of TB was 483 cases per 100,000 individuals in the RMI compared to 2.4 per 100,000 in the US [1, 44]. The alarming rates of TB among Marshallese people have been associated with multiple factors. A shift to highly processed, imported foods have resulted in high rates of malnutrition, obesity, and diabetes and, consequently, increased susceptibility to TB [45, 46]. Moreover, overcrowding and poverty in cities exacerbates TB transmission. There are ~ 30,000 Marshallese immigrants residing in the US, with the largest communities in Arkansas, Hawaii, Oregon, Washington, California, and Oklahoma [47]. Clinicians working in areas with large Marshallese populations should be aware of the high incidence of TB to provide appropriate care [48].

This case demonstrates an indolent and non-specific presentation of disseminated TB. Understanding risk factors for TB, such as recent pregnancy and immigration from TB-endemic regions, are important for early diagnosis and management of TB.

## Data Availability

Not applicable.

## Abbreviations

AFB: Acid-fast bacteria
CSF: Cerebrospinal fluid
CT: Computed tomography
D&C: Dilation and curettage
ETB: Extrapulmonary tuberculosis
FDA: Food and Drug Administration
LP: Lumbar puncture
MRI: Magnetic resonance imaging
NAAT: Nucleic-acid amplification test
PCR: Polymerase chain reaction
PJP: *Pneumocystis jiroveci* pneumonia
PR: Paradoxical reaction
RMI: Republic of the Marshall Island
RIPE: Rifampin, isoniazid, pyrazinamide, ethambutol
TB: Tuberculosis
TBM: Tuberculosis meningitis
US: United States
